# Depression, anxiety, and stress among USMLE aspirants: a cross-sectional survey

**DOI:** 10.1097/MS9.0000000000002985

**Published:** 2025-02-07

**Authors:** Qaisar Ali Khan, Naod F. Belay, Bader Semakieh, Marium Aisha, Fahd Zafar Khan, Elvan Wiyarta, Hoor Ul Ain, Laila Hassan, Hamza Ali Khan, Ravina Verma

**Affiliations:** a Khyber Teaching Hospital MTI KTH, Peshawar, Pakistan; b Michigan State University, East Lansing, Michigan, USA; c Arkansas College of Osteopathic Medicine, Fort Smith, Arkansas, USA; d Bhatti Hospital and Maternity Home, Sukkar, Pakistan; e Department of Neurology, Faculty of Medicine Universitas Indonesia, Cipto Mangunkusumo National Hospital, Jakarta, Indonesia; f Jinnah Medical College, Peshawar, Pakistan; g Lady Reading Hospital, Peshawar, Pakistan; h St. Georges University School of Medicine, True Blue, True Blue, Grenada

**Keywords:** anxiety, depression, mental health, stress management, USMLE

## Abstract

**Background:**

The United States Medical Licensure Examination (USMLE) represents a critical step for medical licensure in the United States, requiring extensive preparation that can lead to significant mental health challenges among aspirants. This study aims to explore the prevalence and contributing factors of depression, anxiety, and stress among USMLE aspirants.

**Methods:**

This cross-sectional survey investigates the prevalence and contributing factors of depression, anxiety, and stress among USMLE aspirants. We deployed a detailed online and paper-based questionnaire targeting a diverse pool of 321 medical students and graduates globally. The survey incorporated validated scales such as the Perceived Stress Scale, Generalized Anxiety Disorder 7-item scale, and Patient Health Questionnaire-9 to evaluate mental health statuses. Data analysis was conducted using SPSS, focusing on demographic correlations and mental health outcomes.

**Results:**

A total of 321 participants’ data were collected, out of whom 51.1% (*n* = 164) were male and 48.9% (*n* = 157) were female. The majority, 91.3% (*n* = 293), were International Medical Graduates (IMG), while 8.7% (*n* = 28) were American Medical Graduates (AMG). Educational status results show that 38.6% (*n* = 124) were undergraduates and 61.4% (*n* = 197) were graduates. Participants were interviewed through a validated questionnaire, and 75.1 % (*n* = 241) had depression, 71.96% (*n* = 231) had anxiety, 4.98% (*n* = 16) had low stress, 71.9% (*n* = 231) had moderate, and 20.5% (*n* = 66) had severe perceived stress.

**Conclusion:**

Our study reveals a high prevalence of depression, anxiety, and stress among USMLE aspirants, with significant variations across demographic subgroups and chosen medical specialties.

**HIGHLIGHTS**

1. This study highlights a high prevalence of depression, anxiety, and stress among USMLE aspirants, as demonstrated by the systematic use of validated instruments such as the Patient Health Questionnaire-9, Generalized Anxiety Disorder 7-item scale, and Perceived Stress Scale.
2. The data demonstrate significant psychological costs across demographic subgroups and medical specialties, emphasizing the varied problems that individuals encounter when negotiating the demanding USMLE program.
3. These mental health difficulties, exacerbated by the intense preparation required for the USMLE, necessitate a strategic emphasis on comprehensive support networks.
4. Addressing these difficulties is critical for aspirants’ well-being and the larger medical community, as it ensures a resilient future workforce capable of providing high-quality treatment.
5. The study’s findings on the mental health landscape among USMLE aspirants open the way for targeted treatments and legislation to mitigate these stressors’ impact, establishing a more supportive atmosphere for medical students and graduates as they begin their professional careers.

## Introduction

The United States Medical Licensing Examination (USMLE) is a licensing assessment mandatory for all allopathic medical students transitioning from student to intern to resident physician. Graduates of accredited osteopathic medical schools may also use the USMLE to meet licensure requirements in most jurisdictions. This three-step examination certifies that successful candidates have the minimum basic and clinical knowledge and clinical skills necessary for the unsupervised practice of medicine in the United States^[1,2]^. The USMLE program is governed by the Federation of State Medical Boards (FSMB), the National Board of Medical Examiners (NBME), the Educational Commission for Foreign Medical Graduates (ECFMG), and the public. The FSMB represents the state medical and osteopathic boards in the United States. In most jurisdictions, FSMB supports all the boards in protecting public health and patient safety through proper licensing, physician regulation and discipline, and other health care professionals^[3]^. NBME provides a variety of quality assessments and educational services for students, professionals, and medical institutions. These assessment tools are used in medical education, licensure, and certification^[4]^. The ECFMG is a private, nonprofit, nongovernmental organization that is authorized by federal regulations to serve as the certifying agency for international medical graduates (IMGs) entering the US physician workforce as trainees in graduate medical education (GME)^[5]^. ECFMG Certification is the standard for evaluating the qualifications of IMGs entering the US health care system. The Accreditation Council for Graduate Medical Education (ACGME) requires IMGs who enter ACGME-accredited residency or fellowship programs to be certified by ECFMG^[6]^.

For Step 1, Step 2, CK, and Step 3, which are the computer-based testing (CBT) components of the USMLE program, test scheduling and delivery are provided by Prometric^[7]^. These CBT examinations are currently administered at more than 345 US/Canadian Prometric test centers and approximately 100 international Prometric test centers. The Step 1 and Step 2 CK examinations are administered worldwide; the Step 3 examination is administered only in the United States^[3]^. In 2022, 91% of US and Canadian medical students and graduates, as well as 71% of foreign medical graduates (IMGs), passed Step 1; for USMLE Step 2 CK, 98% of US and Canadian medical students and grads passed, while 86% of IMGs did^[8,9]^. Similarly, 97% of US and Canadian medical students and graduates passed the USMLE Step 3, whereas 89% of IMGs did^[9]^. In addition, the NRMP will offer 36,277 first-year residency jobs in 2023^[9]^.

The USMLE is not only a rigorous academic test but also a deeply demanding process that requires sustained mental resilience. Each step presents intense intellectual and psychological challenges, which necessitate an unwavering focus, mastery over extensive medical knowledge, and constant self-motivation under competitive conditions. This relentless pressure has made stress, anxiety, and depression prevalent among USMLE aspirants, as the stakes are high, and the fear of failure can have long-lasting implications on career paths. As a result, this challenging pathway often leads aspirants to face chronic stress, which is characterized by symptoms of anxiety, depression, and burnout.

Understanding and addressing these mental health concerns is essential, not only to safeguard the well-being of these future health care professionals but also to prevent burnout and mental health deterioration that may affect the quality of care they are able to provide to patients in the future. The long-term implications of untreated mental health issues in medical trainees can lead to decreased productivity, impaired decision-making, and, ultimately, a decline in patient care standards. Additionally, physicians who enter practice with unresolved mental health issues may find it challenging to manage the demanding nature of the profession effectively.

Given the high stakes, this study aims to explore the prevalence of stress, anxiety, and depression among USMLE aspirants and to examine the various factors that contribute to these mental health challenges. By identifying these stressors, it is hoped that support mechanisms can be implemented to alleviate mental health burdens, ensuring a healthier journey through medical training and better equipping future physicians to provide optimal patient care.

This study highlights the prevalence of stress, anxiety, and depression experienced by USMLE aspirants, as well as the factors that contribute to these challenges.

## Methods

### Study design and sample size

This is a cross-sectional online survey to assess the prevalence of stress, anxiety, depression, and their contributing factors. A total of 321 participants participated in the study. The sample size calculated using the OpenEPI sample size calculator was 273, taking the anticipated frequency of 22%, margin of error of 5%, and 95% confidence interval.

### Data collection and methodology

The convenience sampling method was applied. Participants were recruited from social media platforms (Reddit, LinkedIn, Facebook, X, Instagram, and WhatsApp) made for the guidance of USMLE aspirants. Responses were also collected on-site from two medical schools in Pakistan and the United States of America. The survey was hosted on Google Forms and was widely distributed on these platforms. Following the Helsinki Declaration, records of all participants were checked, and eligible participants were included in the study based on the inclusion criteria. A structured questionnaire was developed consisting of demographic questions (e.g., gender, ethnicity, and educational background), standardized scales for assessing stress (e.g., Perceived Stress Scale), anxiety (e.g., Generalized Anxiety Disorder 7-item scale), and depression (e.g., Patient Health Questionnaire-9), and questions related to the problems they are facing during their USMLE journey and the chosen specialty^[10-12]^. The survey was accessible to collect the responses from 20 January 2023 to 1 December 2023. Participants were encouraged to complete the survey in one setting to preserve the data integrity. The work has been done following the Strengthening the Reporting of Cohort, cross-sectional, and case-control Studies in Surgery (STROCSS) guidelines^[13]^.

#### Inclusion criteria

1. Individuals currently preparing for the USMLE exams (Step 1, Step 2 CK, or Step 3).
2. Recent USMLE aspirants who have completed any of the USMLE exams within the last 6 months.
3. Participants aged 20–40 years.
4. Participants who provided informed consent to participate in the study.
5. International Medical Graduates (IMGs) and American Medical Graduates (AMGs).

#### Exclusion criteria

1. Individuals not actively preparing for or recently completed any USMLE exams.
2. Participants under the age of 20 or over 40.
3. Individuals who did not provide informed consent.
4. Participants who are not graduates or students of accredited medical schools.
5. Respondents with incomplete survey responses. [Incomplete surveys were handled as follows: responses with substantial missing data were excluded from the analysis to maintain data integrity and accuracy. Specifically, if a respondent had leftover 20% of the survey questions unanswered, their response was excluded entirely. For surveys with minor missing data (less than 20%), an imputation method was used to manage isolated missing values. The mean imputation method was applied to continuous variables, while mode imputation was used for categorical variables.]

### Statistical analysis

All data are transferred from Google Forms to Excel Sheets. Quantitative analysis was performed using SPSS version 20 for Windows. Descriptive analysis was employed to characterize the participant’s demographic information, such as age, gender, marital status, graduation status, educational background, working status, preparation stage, and specialty choice. Cross tabulations chi-square test was applied to explore the association between all the demographic variables vs. depression, anxiety, and stress levels. A *P* value <0.05 was considered statistically significant.

## Results

Most of the participants interviewed in this survey were male, accounting for 51.1% (*n* = 164), with females representing 48.9% (*n* = 157). The respondents predominantly fell into two age groups, with the larger number, 88.8% (*n* = 285), in the 20–30 age group, and the remainder, 11.2% (*n* = 36), in the 30–40 age group. A significant portion of the respondents, 83.8% (*n* = 269), were single, 13.7% (*n* = 44) were married, 1.2% (*n* = 4) were in a relationship, and 1.2% (*n* = 4) were in a common-law partnership. In terms of medical education background, 91.3% (*n* = 293) were International Medical Graduates (IMGs), while 8.7% (*n* = 28) were American Medical Graduates (AMGs). Regarding educational status, 38.6% (*n* = 124) were undergraduates, and 61.4% (*n* = 197) were graduates. Detailed demographic information of the participants is provided in Table 1.

**Table 1:** Participants’ demographic characteristics

Variables	Number (*n*)	Percentage
Gender		
Male	164	51.1%
Female	157	48.9%
Age		
20-30 years	285	88.8%
30-40 years	36	11.2%
Marital status		
Single	269	83.8%
Married	44	13.7%
In a relationship	4	1.2%
Common law	4	1.2%
Graduation status		
AMG’s	28	8.7%
IMG’s	293	91.3%
Educational status		
Undergraduate	124	38.6%
Graduated	197	61.4%
Study year (for undergraduates)		
First year	24	7.5%
Second year	4	1.2%
Third year	32	10.0%
Fourth year	44	13.7%
Final year	20	6.2%
Years of graduation (for postgraduates)		
Less than 5 years	169	52.6%
More than 5 years	28	8.7%
Working status (for postgraduates)		
Yes	117	36.4%
No	204	63.6%
Stage of preparation		
Not taken any USMLE stjpg exam	116	36.1%
Taken one USMLE stjpg exam	108	33.6%
Taken two of USMLE stjpg exam	32	10.0%
ECFMG certified	65	20.2%

**Figure 1.**
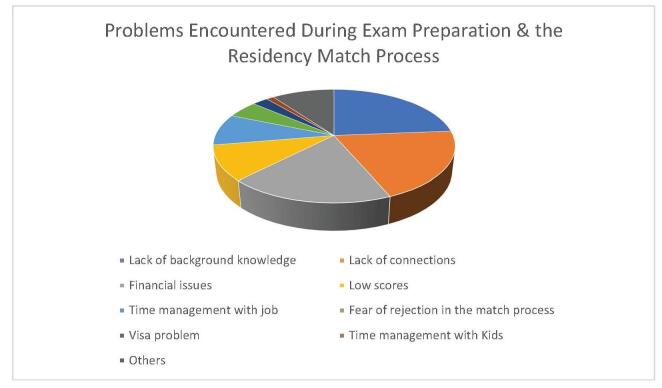
Showing the prevalence of various problems encountered by the respondents.

**Figure 2.**
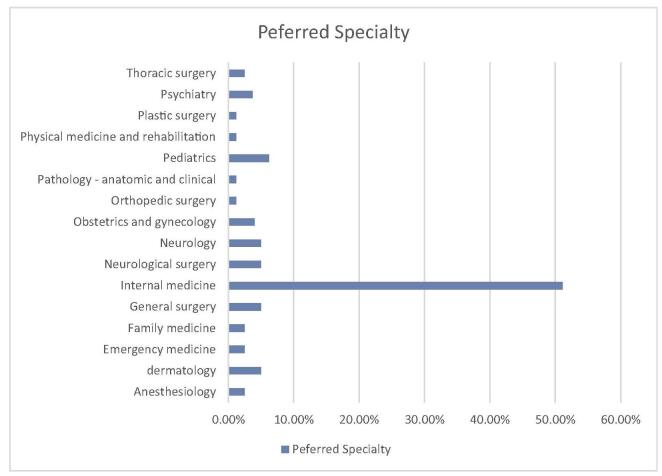
Showing the preferred specialty of the respondents.

Interviews about their challenges during exam preparation or the residency match process revealed that 23.7% (*n* = 76) experienced a lack of background knowledge, 19.9% (*n* = 64) faced a lack of connections for the match process in the United States, 18.7% (*n* = 60) encountered financial issues, 10% (*n* = 32) had low scores, 9% (*n* = 29) reported difficulty in time management with a job, followed by fear of rejection during the match process 5% (*n* = 16), visa problems 2.5% (*n* = 8), and time management with kids 1.2% (*n* = 4), as depicted in Figure 1. Further inquiries about their preferred specialty applications are shown in Figure 2. Participants were also interviewed using the PHQ-9 Questionnaire for depression, the Generalized Anxiety Disorder 7-item (GAD-7) scale for anxiety, and the Perceived Stress Scale for stress assessment.

A significant proportion, 75.1% (*n* = 241), were found to have depression, classified into mild, moderate, moderately severe, and severe categories. The prevalence of depression showed a significant difference between males and females (*P* = 0.001), with the majority of males falling into the mild category (17.4%, *n* = 56), and a more evenly distributed severity pattern among females. Similar distribution trends across depression severity levels were observed in both age groups (*P* = 0.381). A detailed subgroup analysis of depression in terms of marital status, graduation year, year of study, participants’ medical school country, and working status is provided in Table 2. The analysis indicated a higher prevalence of depression among those who lacked background knowledge for preparation (*n* = 52), followed by those who lacked connections in the United States for the match process (*n* = 48) and had financial issues (*n* = 40).

**Table 2:** Prevalence of depression based on the PHQ-9 Questionnaire and the subgroup analysis

Variable	None-minimal	Having depression	Mild	Moderate	Moderately severe	Severe	*P* value
Gender							
Male	60	104	56	44	04	0	0.001
Female	20	137	69	24	24	20	
Age							
20-30 years	72	213	109	64	24	16	0.381
30-40 years	8	28	16	4	4	4	
Marital status							
Single	68	201	97	56	28	20	0.001
Married	12	32	28	4	0	0	
In relationship	0	4	0	4	0	0	
Common law	0	4	0	4	0	0	
AMGs vs IMGs							
AMGs	12	16	8	4	0	4	0.023
IMGs	68	225	117	64	28	16	
Graduation status							
Undergraduate	40	84	40	36	0	8	0.001
Graduated	40	147	85	32	28	12	
Year of study							
First year	0	24	8	16	0	0	0.001
Second year	4	0	0	0	0	0	
Third year	12	20	16	4	0	0	
Fourth year	24	20	12	8	0	0	
Final year	0	20	4	8	0	8	
Year of graduation							
Less than 5 years	32	137	69	28	28	12	0.001
More than 5 years	8	20	16	4	0	0	
Working status							
Yes	28	89	49	16	20	4	0.001
No	52	152	76	52	8	16	
Stage of preparation							
Not taken any stjpg exam	36	80	28	40	4	8	0.001
Taken only one stjpg exam	20	88	48	24	12	4	
Taken two stjpg exams	8	24	8	4	8	4	
ECFMG certified	16	49	41	0	4	4	

For anxiety, assessed using the GAD-7 scale, 71.96% (*n* = 231) of participants had anxiety, who were further categorized into mild, moderate, and severe. There was a statistically significant (*P* < 0.05) difference in the prevalence of anxiety between males and females. Detailed prevalence and subgroup analysis based on age, marital status, graduation year, year of studying, participants’ medical school country, and working status are presented in Table 3. The prevalence of anxiety was notably higher among those lacking background knowledge for preparation (*n* = 54), followed by those lacking US connections for the match process (*n* = 46) and those facing financial issues (*n* = 42).

**Table 3:** Prevalence of anxiety based on the generalized anxiety disorder 7-item scale and the subgroup analysis

Variable	None – minimal	Having anxiety	Mild	Moderate	Severe	*P* value
Gender						
Male	66	98	66	20	12	0.001
Female	24	133	51	40	42	
Age						
20-30 years	84	201	97	54	50	0.078
30-40 years	6	30	20	6	4	
Marital status						
Single	82	187	79	54	54	0.001
Married	6	38	36	2	0	
In relationship	0	4	0	4	0	
Common law	2	2	2	0	0	
AMGs vs IMGs						
AMGs	12	16	8	8	0	0.021
IMGs	78	215	109	52	54	
Graduation status						
Undergraduate	52	72	22	26	24	0.001
Graduated	38	159	95	34	30	
Year of study						
First year	2	22	6	12	4	0.001
Second year	4	0	0	0	0	
Third year	12	20	6	2	12	
Fourth year	34	10	6	4	0	
Final year	0	20	4	8	8	
Year of graduation						
Less than 5 years	36	133	73	30	30	0.001
More than 5 years	2	26	22	4	0	
Working status						
Yes	16	101	59	28	14	0.001
No	74	130	58	32	40	
Stage of preparation						
Not taken any stjpg exam	44	72	26	30	16	0.001
Taken only one stjpg exam	20	88	48	14	26	
Taken two stjpg exams	6	26	12	6	8	
ECFMG certified	20	45	31	10	4	

**Figure 3.**
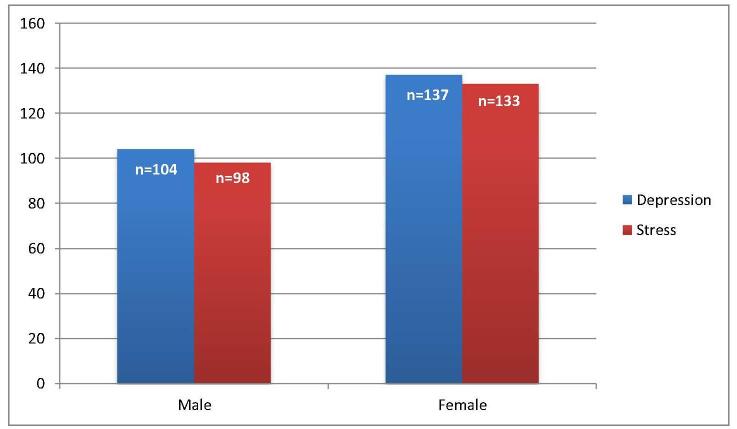
Comparison of depression and anxiety based on gender.

**Figure 4.**
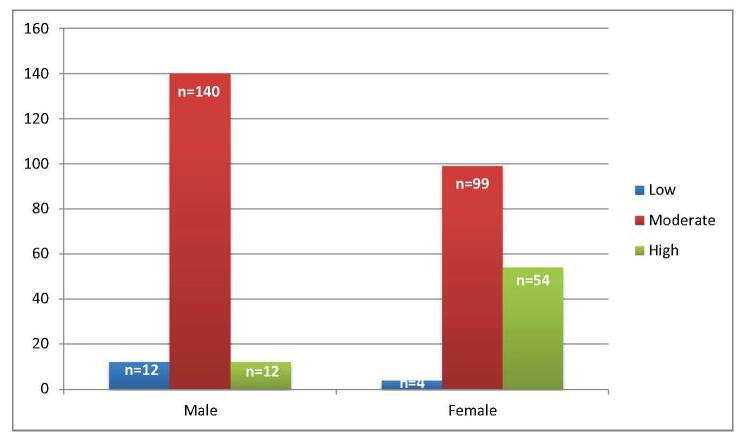
Showing severity level of stress based on gender among USMLE aspirants.

Regarding stress levels, assessed using the Perceived Stress Scale, a small fraction, 16 participants, reported low stress, whereas a substantial number, 239 participants, experienced moderate stress, and 66 reported high perceived stress. A statistically significant gender difference in stress levels was observed (*P* = 0.001), with a larger proportion of male respondents reporting moderate stress. The subgroup analysis of stress, presented in Table 4, showed a higher prevalence among individuals lacking background knowledge for preparation (*n* = 56), followed by those with financial issues (*n* = 52) and those lacking US connections for the match process (*n* = 42). Figures 3 and 4 show a comparison of overall depression, anxiety, and level of stress based on gender.

**Table 4:** Prevalence of stress based on the perceived stress scale and the subgroup analysis

Variable	Low	Moderate	High	*P* value
Gender				
Male	12	140	12	0.001
Female	4	99	54	
Age				
20-30 years	16	213	56	0.215
30-40 years	0	26	10	
Marital status				
Single	16	197	56	0.002
Married	0	38	6	
In relationship	0	0	4	
Common law	0	4	0	
AMGs vs IMGs				
AMGs	10	14	4	0.001
IMGs	6	225	62	
Graduation status				
Undergraduate	6	94	24	0.904
Graduated	10	145	42	
Year of study				
First year	0	16	8	0.001
Second year	0	4	0	
Third year	6	22	4	
Fourth year	0	40	4	
Final year	0	12	8	
Year of graduation				
Less than 5 years	10	123	36	0.736
More than 5 years	0	22	6	
Working status				
Yes	6	89	22	0.840
No	10	150	44	
Stage of preparation				
Not taken any stjpg exam	0	90	26	0.001
Taken only one stjpg exam	10	72	26	
Taken two stjpg exams	4	18	10	
ECFMG certified	2	59	4	

**Figure 5.**
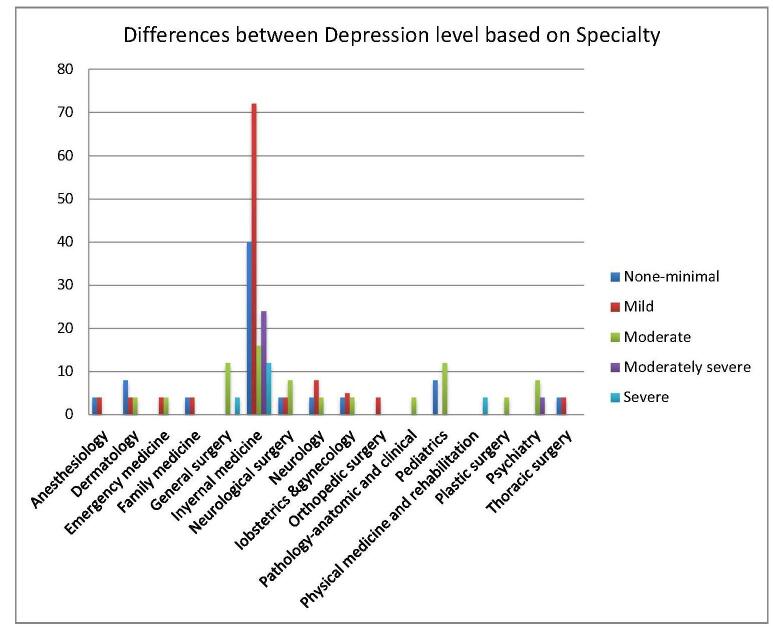
Shows the level of depression based on specialty among USMLE aspirants.

**Figure 6.**
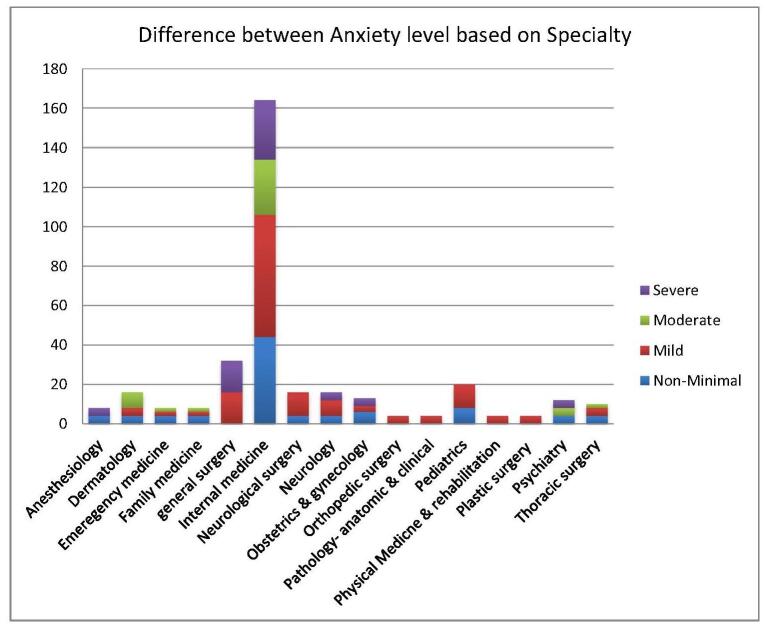
Shows the level of anxiety based on specialty among USMLE aspirants.

**Figure 7.**
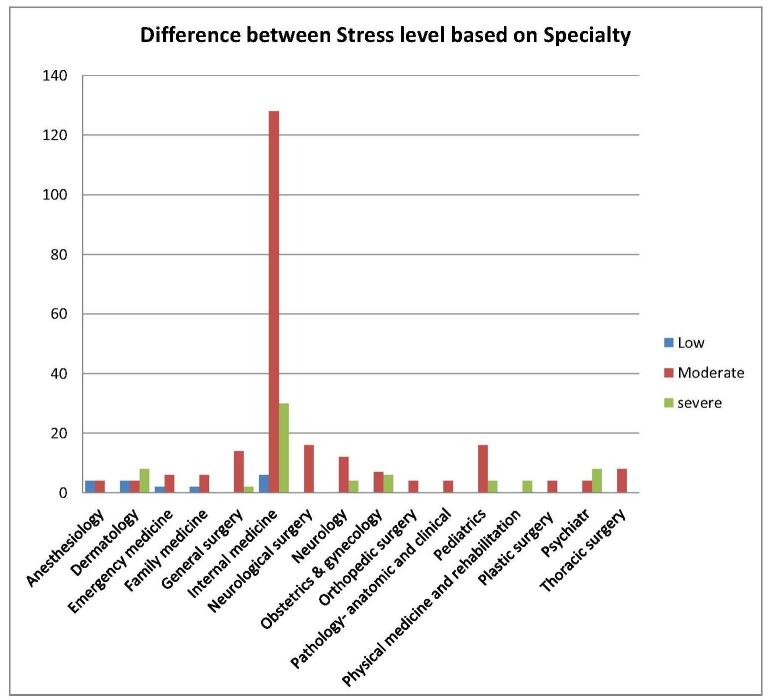
Shows the level of stress based on specialty among USMLE aspirants.

Table 5 illustrates the statistically significant differences (*P* < 0.05) in depression, anxiety, and stress levels based on the specialty chosen, emphasizing the nuanced impacts these factors have on USMLE aspirants’ mental health. Additionally, Figures 5, 6, and 7 offer graphical representations of these findings, providing a detailed depiction of the severity levels for each factor.

**Table 5:** Difference between depression, anxiety, and stress based on specialty

Depression	None-minimal	Having depression	Mild	Moderate	Moderately severe	Severe	*P* value
Anesthesiology	4	4	4	0	0	0	0.001
Dermatology	8	8	4	4	0	0	
Emergency medicine	0	8	4	4	0	0	
Family medicine	4	4	4	0	0	0	
General surgery	0	16	0	12	0	4	
Internal medicine	40	124	72	16	24	12	
Neurological surgery	4	12	4	8	0	0	
Neurology	4	12	8	4	0	0	
Obstetrics and gynecology	4	9	5	4	0	0	
Orthopedic surgery	0	4	4	0	0	0	
Pathology – anatomic and clinical	0	4	0	4	0	0	
Pediatrics	8	12	12	0	0	0	
Physical medicine and rehabilitation	0	4	0	0	0	4	
Plastic surgery	0	4	0	4	0	0	
Psychiatry	0	12	0	8	4	0	
Thoracic surgery	4	4	4	0	0	0	
Total	80	241	125	68	28	20	
**Anxiety**	**None-minimal**	**Having anxiety**	**Mild**	**Moderate**	**Severe**		***P* value**
Anesthesiology	4	4	0	0	4		0.001
Dermatology	4	12	4	8	0		
Emergency medicine	4	4	2	2	0		
Family medicine	4	4	4	0	0		
General surgery	0	16	0	16	0		
Internal medicine	44	120	62	28	30		
Neurological surgery	4	12	12	0	0		
Neurology	4	12	8	0	4		
Obstetrics and gynecology	6	7	3	0	4		
Orthopedic surgery	0	4	4	0	0		
Pathology – anatomic and clinical	0	4	4	0	0		
Pediatrics	8	12	12	0	0		
Physical medicine and rehabilitation	0	4	0	0	4		
Plastic surgery	0	4	0	0	4		
Psychiatry	4	8	0	4	4		
Thoracic surgery	4	4	2	2	0		
Total	90	231	117	60	54		
**Stress**	**Low**	**Moderate**	**High**				***P* value**
Anesthesiology	4	4	0				0.001
Dermatology	4	4	8				
Emergency medicine	2	6	0				
Family medicine	0	8	0				
General surgery	0	14	2				
Internal medicine	6	128	30				
Neurological surgery	0	16	0				
Neurology	0	12	4				
Obstetrics and gynecology	0	7	6				
Orthopedic surgery	0	4	0				
Pathology – anatomic and clinical	0	4	0				
Pediatrics	0	16	4				
Physical medicine and rehabilitation	0	0	4				
Plastic surgery	0	4	0				
Psychiatry	0	4	8				
Thoracic surgery	0	8	0				
Total	16	239	66				

## Discussion

This study examines variations in the prevalence and intensities of depression, anxiety, and stress across different demographic categories of USMLE candidates, taking into account differences in gender, age, marital status, and educational background. The main obstacles highlighted for USMLE candidates are a lack of fundamental knowledge, inadequate social assistance, and financial burden. Various potential factors that contribute to mental health concerns among USMLE hopefuls have been identified, such as academic pressures, financial stress, and the high stakes involved with the USMLE tests. Our study shows a high prevalence of depression, anxiety, and stress among USMLE aspirants. Depression rates varied greatly across a number of parameters, aside from age. The demands of preparation and the pressure to perform well combine to create a high-stress atmosphere that can affect mental health. There is a lot of research on the USMLE tests, but very few studies have looked at the way they affect the well-being of USMLE candidates. Students feel a lot of stress and anxiety when taking the Step 1 exam. Many aspirants feel that their academic program inadequately matches the exam’s criteria; they spend a lot of time and money on commercial review courses in an attempt to find structure and order. Studies show that these classes do not result in improved scores. Whether commercial courses offer students real benefits or just increase the financial and psychological strain of studying for exams is a topic of constant debate.

Commercial test preparation courses, books, and question banks have increased in popularity because of the high-stakes nature of the USMLE Step 1 Commercial courses do not improve Step 1 scores for a large group of students, according to studies. Peer-designed review courses have been associated with better exam performance. In a study, Thadani *et al* showed that the participants taking various board preparation courses did not significantly improve their Step 1 scores. It remains a pressing issue that demands attention, as this inconsistency highlights the need for further exploration into the stress and challenges individuals face preparing for these exams^[10,11]^.

Thus, USMLE exams exemplify a crucial challenge: the congruence between the content of the curriculum and the demands of the exams. The disparity between the content taught to students and the content assessed in tests can intensify feelings of inadequacy and lack of readiness, resulting in increased levels of worry and stress. The development of the curriculum needs to be synchronized with the needs of licensure exams to implement a comprehensive approach to medical education. The goal is to alleviate the psychological strain on students while guaranteeing the excellence of medical education and patient care.

Further, Internal Medicine is a highly sought-after field, receiving a substantial number of applications during the match process, with Family Medicine, Pediatrics, Neurology, and Pathology following closely behind. In 2023, it was reported that 56% of US International Medical Graduates (IMGs) (1051 out of 1877 individuals) and 59% of non-US IMGs (2107 out of 3591 individuals) successfully matched into their preferred specialty of Internal Medicine^[14]^. Additionally, US IMGs and non-US IMGs occupied 12.1% and 27.2% of the positions in Internal Medicine, respectively, highlighting its IMG-friendly nature^[14]^. Data from a study of community-based Internal Medicine residency programs indicated that IMGs outperformed USMGs in self-assessed fatigue, personal growth, and self-esteem scales during their transition to residency^[15]^. The authors attribute these findings to the energy and enthusiasm that foreign graduates bring, despite facing substantial challenges in pursuing US GME training. Additionally, IMGs often exhibit superior medical school performance compared to USMGs enrolled in less popular specialties like Internal Medicine^[15,16]^. Moreover, our data found a strong correlation between mental health outcomes and the choice of medical specialty. Our result shows among the participants those who pursued Internal Medicine showed the highest rates of depression, followed by individuals in General Surgery, Neurosurgery, Neurology, and Pediatrics. A similar trend was observed for anxiety and elevated levels of perceived stress, with applicants in Internal Medicine being particularly affected.

Many participants reported facing significant challenges during the USMLE preparation and match process, inadequate networking for the US match procedure, and financial difficulties. Few studies have examined the results of IMGs’ residency matches. The competition to be matched into residency has increased^[17]^. According to one study, the probabilities of matching were lower when applying to more specializations^[18]^. This result is in line with findings from the NRMP that show applicants who apply to more specialties have a reduced chance of finding a match. Residency programs may perceive applicants with multi-specialty resumes as less dedicated to any one specialty, which could explain this tendency^[18]^. Additionally, applicants aiming for more competitive specialties may apply to less competitive ones as fallback options^[19]^. Moreover, the challenges confronted by IMGs begin before residency application when they must build a highly competitive profile aiming for a barely equal chance of matching their US peers^[20]^. Despite higher United States Medical Licensing Exams scores, IMGs have a lower probability than USMGs of being considered for an interview and subsequently being accepted into a residency program^[21]^. Thus, factors that increase the probability of securing a residency by IMGs include elevated USMLE scores, US clinical experience through hands-on rotations, letters of recommendation from US physicians, and scientific research^[22,23]^. These requirements are stressful and often a cause of depression, especially for IMGs. Among the top 10 nations sending applicants for ECFMG J-1 visa sponsorship are several low- and middle-income countries, where economic constraints pose significant challenges^[24]^. These limitations make it difficult for applicants to participate in hands-on rotations, secure letters of recommendation from US physicians, and familiarize themselves with the US GME and healthcare system conditions, often making these opportunities accessible only to those with ample resources. Many residency programs require or prefer US direct patient care experience, yet not all US teaching hospitals accept IMGs for clinical clerkships^[21,25]^. Those that do often have lengthy application processes, stringent requirements, and high application fees^[25]^.

The IMGs struggle with securing a residency in the United States and the financial burden, including examination fees, USCEs, interview expenses, and visa sponsorship costs, vary widely depending upon individuals’ circumstances. Factors such as dietary preferences, travel arrangements, accommodations, and rescheduling of exams can significantly influence the overall cost, often making it unpredictable. This further contributes to stress, anxiety, and depression levels. USMLE exam preparation can raise overall costs to unpredictable levels. Other factors such as dietary preferences, travel arrangements, accommodations, and rescheduling of exams significantly influence individuals’ budgets^[26]^.

The estimated cost for an international medical graduate (IMG) to complete the USMLE stjpg and secure a residency is $22 735 to $37 685. This includes fees for ECFMG certification, USMLE exam fees, and potential rescheduling costs. Additional expenses for visa sponsorship, ERAS application, travel, accommodation, preparation materials, immunizations, malpractice insurance, health and travel insurance, visa fees, and international flights can also increase the expenses^[26]^. Moreover, commentary on Step 1 indicates that the financial burden of exam preparation exacerbates the learning environment’s challenges, contributing to inequities and maldistribution within the physician workforce^[27]^. Therefore, addressing educational debt has been suggested as a strategy to enhance medical students’ well-being.

Our analysis also revealed a significant disparity between female and male levels of depression, anxiety, and stress, with females experiencing higher severity levels, especially among married IMGs. This disparity may be attributed to the added pressures women face, including societal expectations, balancing family responsibilities, and potential experiences of gender bias within the medical field. This finding aligns with previous research that has explored the impact of various demographic variables on USMLE performance^[28]^. For example, one study indicated that, after accounting for pre-matriculation measures, men slightly outperformed women on Step 1, with undergraduate science GPA being more predictive of Step 1 performance for women. However, the same research team found that, in a large sample of examinees, women outperformed men in most content areas on the USMLE Step 2 CK exam, and this gender-related difference became more pronounced when controlling for Step 1 scores^[29]^. This suggests the fact that despite women facing more obstacles in their early years, they often outperform as they progress. According to similar studies in the field, the stress associated with USMLE tests is consistent with more general findings in medical education research. Thus, offering focused assistance for reducing early strains is required. An investigation conducted by Dyrbye *et al*^[30]^ regarding the mental health of medical students in the United States revealed that the occurrence of burnout and depressive symptoms among medical students was notably greater compared to the general population. As found in our results, this is especially true for those students preparing for the USMLE. The anxiety associated with USMLE preparation is similar to the stress experienced due to the great importance of passing the exam, as it not only determines licensing but also affects the chances of securing a residency placement^[31]^.

As data from earlier research indicates a higher prevalence and incidence rate of anxiety and depression among medical students than the general population. These mental health issues can have a detrimental effect on academic achievement and, in certain situations, result in substance abuse as a coping strategy. A “hidden curriculum” that promotes skepticism is one of these difficulties, along with extreme academic pressure, a tremendous workload, financial strain, chronic sleep deprivation, exposure to patient suffering and death, and incidents of student abuse. Cultural backgrounds, family educational attainment, and non-academic demands are other factors^[32-34]^. The relationship between personal resilience, coping techniques, and external support systems should be further investigated to uncover factors that can reduce the psychological effects of USMLE preparation. This comprehension could guide the creation of comprehensive support systems that cater to the educational and psychological requirements of those striving for success^[35]^. Social media and other digital platforms are greatly impacting medical students. They frequently use these platforms for social interaction as well as academic resources. Therefore, additional research is necessary to determine how social media and digital platforms affect USMLE applicants’ stress levels^[36]^. Our study also shows significant high disparities in the prevalence of anxiety and depression among both genders, which emphasizes the necessity of gender-specific needs and mental health assistance. Support services can be more successfully used to promote well-being and lessen mental health burdens by being customized to address the particular difficulties faced by male and female candidates^[37]^.

It is well established that emotions of loneliness and competition are linked to mental health issues, thus creating a supportive environment among USMLE candidates through programs like peer mentorship, cooperative study groups, and wellness-focused seminars can significantly lessen these feelings. Furthermore, creating an atmosphere in medical schools that values emotional transparency and openness will enable students to ask for assistance without worrying about stigma or condemnation. This can be accomplished by institutional rules that normalize discussions about mental health issues and place an emphasis on psychological safety. It can also be advantageous to implement focused educational programs that give students useful study strategies for the USMLE. Carle Illinois College of Medicine launched a unique course to improve students’ preparatory experience by attending to their academic and mental health needs. The participants experienced notable benefits^[38,39]^.

The study has several limitations. First, using a simple convenience sampling might have introduced selection bias, limiting the generalizability of the findings to the entire population of USMLE aspirants. Second, reliance on self-reported data may lead to response bias and inaccuracies, as participants might overestimate or underestimate their stress, anxiety, and depression levels. The cross-sectional design captures data at a single point in time, limiting the ability to establish causality between USMLE preparation and mental health outcomes. Additionally, the study collected limited demographic information; a more comprehensive set of demographic and socio-economic data would have provided a better understanding of the factors influencing mental health. Finally, conducting the survey online might exclude those with limited internet access or those less likely to engage in social media and online platforms, potentially leading to an unequal presentation of certain groups. The strength of the study includes a large sample size, and the precision and consistency of the scales are guaranteed by the use of validated and standardized instruments such as the Perceived Stress Scale, GAD-7, and PHQ-9. The significance of the study to a wide range of participants is increased by incorporating both AMGs and IMGs. Moreover, the thorough examination of mental health problems across diverse demographic groups reveals important information about groups that could be particularly vulnerable. Finally, considering the demanding nature of the USMLE tests and the increasing emphasis on mental health in medical education, this study tackles a pressing topic and makes significant contributions to the body of knowledge already in existence.

## Conclusion

The data demonstrate significant psychological costs across demographic subgroups and medical specialties, emphasizing the varied problems that individuals encounter when negotiating the demanding USMLE program. These mental health difficulties, exacerbated by the intense preparation required for the USMLE, necessitate a strategic emphasis on comprehensive support networks. Addressing these difficulties is critical for aspirants’ well-being and the larger medical community, as it ensures a resilient future workforce capable of providing high-quality treatment. Furthermore, a prospective study should be done to explore intervention strategies such as institutional support, mental health services, peer networks, or coping mechanisms for getting a casual conclusion.

## Data Availability

Data can be available upon reasonable request to the corresponding author.

## Research registration unique identifying number (UIN)

Research Registry (researchregistry10491).
